# Adapting PCIT-Health for Telehealth Delivery: A Case Study

**DOI:** 10.3390/ijerph19148352

**Published:** 2022-07-08

**Authors:** Sarah E. Domoff, Mikaela M. Overton, Aubrey L. Borgen, Larissa N. Niec

**Affiliations:** 1 Department of Psychology, Central Michigan University, Mount Pleasant, MI 48858, USA; overt1mm@cmich.edu (M.M.O.); borge1al@cmich.edu (A.L.B.); niec1l@cmich.edu (L.N.N.); 2 Center for Children, Families, and Communities, Central Michigan University, Mount Pleasant, MI 48858, USA

**Keywords:** PCIT, telehealth, obesity, problematic media use, feeding

## Abstract

During the COVID-19 pandemic, children’s physical health and access to mental health resources have been two critical concerns. Parent-Child Interaction Therapy-Health (PCIT-Health) is a treatment model aimed at helping parents manage children’s general behavior and their behavior in obesogenic contexts (screen time and mealtime). Due to social distancing guidelines, PCIT-Health was adapted for remote delivery through video conferencing. In this article, we describe the experience of implementing virtual PCIT-Health with a family. The family’s progress through treatment is described, along with the challenges associated with remote service delivery and how those challenges were addressed. Progress through treatment was measured with questionnaires administered to caregivers and with observational measures of parent-child interactions. The results from these measures indicate that caregivers experienced a reduction in stress and improvements in their child’s behavior after PCIT-Health completion. They also reported engaging in healthier management of their child’s screen time and mealtime behaviors. As coded from observational assessments, parents increased their use of positive parenting practices. Telehealth-delivered PCIT-Health is a promising treatment modality for increasing parenting skills and improving child behavior.

## 1. Introduction

Childhood obesity is a critical public health issue. As of 2017–2018, 19.3% of children and adolescents in the United States were identified as meeting criteria for obesity (i.e., having a body mass index (BMI) at or above the 95th percentile) [1]. This prevalence has increased drastically among US youth, as 19 years ago 13.9% of children and adolescents met criteria for obesity, and 50 years ago only 5.2% of children and adolescents met those criteria [2]. Childhood obesity increases individuals’ risk for serious, negative health outcomes such as high blood pressure, type 2 diabetes, breathing problems, and joint problems [3,4,5], and tends to be a fairly stable issue, as experiencing obesity in childhood increases the likelihood of experiencing obesity as an adolescent and adult [6].

Because of these potentially negative, long-term impacts of childhood obesity, it is essential to identify how children’s risk for obesity can be reduced through modification of parenting practices. Existing research suggests that how parents interact with their children around mealtimes can reduce or increase their risk for obesity. For example, feeding practices that reduce risk include promoting children’s autonomy during mealtimes by allowing them to practice making food choices and helping them to recognize when they are full [7]. Parents can increase their children’s appropriate and healthy mealtime behaviors by describing what the child is doing or praising this behavior [8]. Parents can further promote autonomy and healthy behaviors by providing appropriate structure for the mealtime environment, including offering healthy food choices, creating routines, and reducing distractions [9]. Conversely, parents can increase their children’s risk for obesity by decreasing their autonomy during mealtimes, either by pressuring a child to eat more, which can decrease their ability to attend to satiety cues, or by over-restricting unhealthy foods, which can prevent their learning how to eat different types of food in moderation [10,11]. Children’s risk for obesity also increases when parents frequently provide food as a reward for desirable behavior or withhold food as a punishment for undesirable behavior [12].

While parents can have an impact on their children’s lifelong eating habits, they also influence how children engage with screens, another risk factor for obesity. Currently, one of the most prevalent sedentary activities among children is their use of screen devices, such as television, video game consoles, and smartphones. Parents play an important role in helping children find a healthy balance between screen time and other activities by setting limits on the amount of screen time and identifying when and where screens can be used [13]. To prevent negative impacts from screen time, it is important for parents to limit device use during mealtime and bedtime [14]. Additionally, parents can mitigate the impact of media content by setting up parental controls on devices or being directly involved in their children’s screen time [15].

The COVID-19 pandemic and the subsequent stay-at-home orders heightened the risk for childhood obesity. During the pandemic lockdown mandates, children increased snacking, increased intake of fried foods and desserts, and decreased consumption of fresh food [16]. One study found that 41.4% of families enrolled in a school-based nutrition program (N = 1048) decreased their fruit and vegetable intake [17]. Many families also increased their non-nutritive use of food (i.e., using food for reward or punishment), especially when they were experiencing increased stressors related to the COVID-19 pandemic [17]. In addition to an increase in unhealthy eating practices, many children were prevented from engaging in physical activity due to online schooling and the cancellation of group activities. Since the beginning of lockdown orders, children have experienced significant reductions in structured physical activity and increased sedentary behavior for both school activities and leisure [18]. Because of these lifestyle changes, the rate of BMI increase among children and adolescents has approximately doubled during the pandemic [19].

While risk factors for obesity in children have increased due to the COVID-19 pandemic, some families are experiencing a benefit: increased accessibility to virtual mental health treatment. Over the past decade, considerable effort has been channeled into using technology as a possible solution to overcome health disparities, particularly for those in rural and other under-served communities. Existing research suggests that mental health services delivered through telehealth may have comparable outcomes to in-person services [20]. Telehealth as a delivery format for mental health services significantly increased after the start of the pandemic. In a large sample of US mental health professionals (N = 768), 39% reported providing some telehealth services before the beginning of the COVID-19 pandemic, which increased to 98% during the pandemic [21].

Several well-known parenting programs have been implemented successfully through internet delivery including Triple P (Positive Parenting Program) [22,23], Barkley’s Defiant Child Program [24], Bootcamp for ADHD (BC-ADHD) [25], and Parent–Child Interaction Therapy (PCIT) [26]. These programs were developed specifically to address children’s externalizing behavior problems (e.g., noncompliance, defiance, aggression). Externalizing behaviors that manifest at an early age can develop into longer-term behavior disorders that markedly impact children’s functioning [27,28,29], and behavior parent training (BPT) programs are considered best practice to address externalizing behavior problems in young children [30]. When delivered in a telehealth format, BPT programs demonstrate efficacy in improving parenting skills [26] and reducing child externalizing behaviors [22,23,24] and parent distress [22,23].

One BPT program that has particular potential to address not only externalizing behaviors but also behaviors related to childhood obesity risk is PCIT. PCIT is a well-established, transdiagnostic BPT program for families with children between 2 years old and 6 years, 11 months old [31,32]. The focus of PCIT is the development of nurturing and responsive caregiving within the context of developmentally appropriate limit-setting [31]. PCIT includes two phases: Child-Directed Interaction (CDI) and Parent-Directed Interaction (PDI). During the first phase (CDI), parents acquire child-centered interaction skills and non-confrontational behavior-management skills (e.g., selective attention/differential reinforcement). During the second phase (PDI), parents learn to use effective, consistent limit-setting. The in vivo therapeutic coaching of the parent during interactions with their child is the primary mechanism of skill acquisition in PCIT [33]. Therapists use behaviorally based coaching techniques such as reinforcement, modeling, and shaping to aid in the development of responsive caregiving and healthier interactions. PCIT is a robust, cost-effective intervention with long-lasting effects [34,35,36].

PCIT is uniquely conducive to the telehealth format, making it compatible with the challenges of the pandemic and providing a means of reducing disparities in service access [37,38]. By design, PCIT requires the therapist to observe the parent–child interaction and provide in vivo coaching behind a one-way mirror through a non-invasive earpiece (i.e., the therapist is separated from the family), and the focus on the model is the development of the parent–child relationship, not the therapist–child relationship [39]. Thus, the intervention is not so different from coaching through a web-based platform via a Bluetooth headset in the family’s own home. Internet-based PCIT (I-PCIT) offers comparable therapist contact to that of traditional, in-clinic PCIT and may provide one pathway to overcoming traditional barriers to effective care [37]. In its first randomized control trial, I-PCIT documented improvements in child behaviors, parenting skills, and overall family functioning [26]. For a more in-depth review of telehealth services, see Ros-Demarize et al. for synchronous parenting interventions and Breitenstein et al. for asynchronous parenting interventions [40,41].

Below we describe a case study that illuminates some of the advantages and challenges of delivering an adaptation of PCIT, PCIT-Health, synchronously via the internet. PCIT-Health was developed to reduce children’s obesity risk by teaching parents healthy strategies to manage their children’s behaviors in general contexts and in obesogenic contexts (e.g., relation to screen time and mealtime). We describe the case of Mr. and Mrs. Johnson and their daughter Ellen, a family who participated in the first randomized clinical trial (RCT) measuring the feasibility and efficacy of PCIT-Health as a prevention program.

## 2. Method

### 2.1. Case Description

Mr. and Ms. Johnson contacted a university psychological clinic during the Fall of 2020 to receive services that would help them manage the behavior of Ellen, their 3-year-old daughter. Both Mr. and Ms. Johnson identified their race as White/non-Hispanic. They lived together with their daughter in a rural, midwestern town. To protect the family’s privacy, pseudonyms are used and demographic details are modified.

The primary presenting problems that the Johnsons wished to address included Ellen’s noncompliance and difficulty with emotion regulation. For example, the Johnsons reported that conflict would often arise when Ellen was asked to put away her toys or get ready for bed. She would frequently defy her parents’ requests and throw tantrums that included whining and crying. The Johnsons reported using multiple strategies to manage these behaviors, such as removal of privileges, timeout, and reasoning with Ellen. The Johnsons also reported that Ellen exhibited difficult behaviors during mealtime including leaving the table during the meal and refusing to eat the prepared food. They indicated that Ellen had occasional conflicts with peers (e.g., pushing, not sharing) but stated that 75% of the time Ellen interacted positively and appropriately with other children her age. The Johnsons reported that they had not participated in other parenting interventions over the past year.

In addition to these challenges, the Johnsons also exhibited several strengths. Mr. and Ms. Johnson appeared to have a strong, supportive parenting alliance while both were nurturing and supportive towards Ellen. Prior to beginning the program, the Johnsons expressed the following goals: (1) build a stronger relationship with Ellen, (2) attain parenting skills to handle Ellen’s challenging behaviors, and (3) develop healthier eating habits as a family.

### 2.2. Procedure

Behavioral assessment was integral to measuring the attainment of these goals and occurred before, during, and following the completion of clinic services. As part of a larger Randomized Clinical Trial (RCT; NCT03982511), clinicians administered survey measures and collected observational data from the Johnson family. Most assessments were completed at two time points: pre- and post-intervention. Any exceptions to this timeline are specified below.

#### 2.2.1. Eyberg Child Behavior Inventory (ECBI)

The Eyberg Child Behavior Inventory (ECBI) is a 36-item standardized parent-reported measure of child and adolescent behavioral problems [42]. Parents record the frequency of their children’s problem behaviors on a 7-point scale and specify whether each specific behavior posed a problem for them. Higher scores indicate a higher frequency of problematic behaviors and higher parental concern. Subscale scores were calculated by summing all items. Sample items include “Refuses to obey until threatened with punishment” and “Has temper tantrums”. Research has documented excellent internal consistency across scales (Total Frequency, α = 0.98; Total Satisfaction, α = 0.98) [43]. The ECBI was administered at pre- and post-treatment but also at the transition between each phase of the program.

#### 2.2.2. Psychosocial Strengths Inventory for Children and Adolescents (PSICA)

The Psychosocial Strengths Inventory for Children and Adolescents (PSICA) is a 36-item parent-reported measure of the psychosocial competencies (i.e., affective, attentional, and social competencies) of school-aged children [44]. Parents record the frequency of their children’s prosocial behaviors on a 7-point scale and indicate whether they are satisfied with each behavior. Higher scores indicate a higher frequency of child psychosocial strengths and higher parental satisfaction. Subscale scores were calculated by summing all items. Sample items include “Obeys without threat of punishment” and “Is calm if doesn’t get own way”. Research has documented excellent internal consistency across scales (Total Frequency scale α = 0.97; Total Satisfaction scale KR-20 = 0.95) [44]. The PSICA was administered at pre- and post-treatment as well as weekly throughout the program.

#### 2.2.3. Emotion Regulation Checklist (ERC)

The Emotion Regulation Checklist (ERC) is a 24-item parent-reported measure of the stability, intensity, flexibility, and situational appropriateness of children’s positive and negative emotions [45]. There are two subscales: Emotion Regulation, measuring children’s emotional understanding and empathy, and Lability, measuring children’s lack of flexibility and anger dysregulation. The overall affective regulation score was derived from the sum of all items, which involved reverse scoring some negatively valenced items. Higher scores indicate greater emotional regulation. Sample items include “Is empathic towards others” and “Exhibits wide mood swings”. Both scales have demonstrated acceptable internal consistency (Emotion Regulation, α = 0.83; Lability, α = 0.96) [45].

#### 2.2.4. Sleep Questionnaire

The Sleep Questionnaire used in the current study was adapted from the Children’s Sleep Habit Questionnaire (CSHQ) [46]. This measure assesses children’s sleep behaviors (e.g., sleep resistance, anxiety) and their typical sleep schedule for an average week. Sample items include “My child falls asleep in own bed” and “My child is afraid of sleeping alone”. Some items are reverse scored; higher scores indicate greater sleep difficulties. The CSHQ has shown acceptable internal consistency (α = 0.68 − 0.78) [46].

#### 2.2.5. Child Feeding Questionnaire (CFQ)

The Child Feeding Questionnaire (CFQ) is a 31-item parent-reported measure of parents’ attitudes, beliefs, and practices regarding child feeding [10]. For the purposes of the current program, only two subscales (i.e., 12-items), Restriction (restricting child access to certain foods) and Pressure to Eat (encouraging/pressuring children to consume foods), were administered to parents. Sample items include “I intentionally keep some foods out of my child’s reach” (Restriction) and “My child should always eat all of the food on her plate” (Pressure to Eat). Higher scores indicate greater non-responsive feeding practices (i.e., increased food restriction and increased pressure to eat). Subscale scores were obtained by calculating the means of the items comprising each scale. Both subscales have demonstrated acceptable internal consistency (Restriction, α = 0.73; Pressure to Eat, α = 0.70) [10].

#### 2.2.6. Parental Feeding Style Questionnaire (PFQ)

The Parental Feeding Style Questionnaire (PFQ) is a 27-item parent-reported measure of parental feeding practices. Developed by Wardle et al., the PFQ was based on semi-structured interviews with mothers and clinical and experimental literature describing parental feeding behaviors [47]. In the current study, parents complete a 9-item version of the questionnaire that included items from two subscales: Emotional Feeding and Instrumental Feeding. Sample items from these scales include “I give my child something to eat to make him/her feel better when he/she is upset” (Emotional Feeding) and “I reward my child with something to eat when he/she is well-behaved” (Instrumental Feeding). Subscale scores were obtained by calculating the means of the items comprising each scale. Internal consistency as measured by Cronbach’s alpha (α) has been found to be 0.65 and 0.85 for Emotional Feeding and Instrumental Feeding, respectively [47].

#### 2.2.7. Problematic Media Use Measure-Short Form (PMUM-SF)

The Problematic Media Use Measure-Short Form (PMUM-SF) is a 9-item parent-reported measure of dysregulated media use in children [48]. Parents rate the frequency of their children’s problematic media use on a 5-point Likert scale with higher scores indicating greater problematic screen use. Scale scores were calculated by taking the mean of items. Sample items include “It is hard for my child to stop using screen media” and “My child sneaks using screen media”. The PMUM-SF has demonstrated excellent internal consistency (α = 0.93) [48]. Parents also report on children’s daily screen time across various devices.

#### 2.2.8. Parental Mediation Scale (PMS)

The Parental Mediation Scale (PMS) is a 15-item parent-report measure of parental mediation of children’s television viewing [49]. It has three scales: restrictive mediation (e.g., “How often do you forbid your child to watch certain programs”), instructive mediation (e.g., “How often do you point out why some things actors do are good?”), and social co-viewing (e.g., “How often do you watch together because you both like a program?”). Parents rate the frequency of each mediation behavior on a 4-point Likert scale. Subscale scores were calculated by summing all items, with higher scores illustrating higher levels of mediation for each respective scale. All scales have demonstrated acceptable internal consistency (Restrictive Mediation, α = 0.79; Instructive Mediation, α = 0.80; Social Co-viewing, α = 0.79) [49].

#### 2.2.9. Parenting Stress Index-Short Form (PSI-SF)

The Parenting Stress Index-Short Form (PSI-SF) is a 36-item parent-reported measure of parenting stress relative to the parent-child relationship [50]. It measures three main domains: Parental Distress (e.g., “I often feel that I cannot handle things very well”), Parent–Child Dysfunctional Interaction (e.g., “My child smiles at me less than I expected”), and Difficult Child (e.g., “My child generally wakes up in a bad mood”). All three scales combine to create an overall measure of Total Stress for the parent. Higher scores on all scales indicate greater levels of parental stress. The PSI-SF has demonstrated good internal consistency across scales (Parental Distress, α = 0.90; Parent–Child Dysfunctional Interaction, α = 0.89; Difficult Child, α = 0.88; Total Stress, α = 0.95) [50].

#### 2.2.10. Barriers to Treatment Participation Scale (BTPS)

The Barriers to Treatment Participation Scale (BTPS) is a 44-item parent-reported measure of parental perceived barriers to treatment participation that has demonstrated acceptable internal consistency (α = 0.86) [51]. Parents record how often each respective statement was a barrier to their participation on a 5-point Likert scale. Sample items include “Scheduling of appointment times for the program” and “Transportation (getting a ride, driving, taking a bus) to the clinic for a session” [51]. The total score was calculated by summing all items, with higher scores illustrating the experience of more barriers to participation. The BTPS was administered after the completion of the program.

#### 2.2.11. Abbreviated Acceptability Rating Profile (AARP)

The Abbreviated Acceptability Rating Profile (AARP) was developed as a brief measure of the acceptability of treatment interventions [52]. It is an adaptation of the 15-item Intervention Rating Profile and contains 8 items rated on a 6-point Likert scale. Sample items include “This is an acceptable program for children’s behavior” and “Overall, the program would help children”. Scaled scores were calculated by summing all items, and higher scores illustrate greater treatment acceptability [53]. It has demonstrated excellent internal consistency (α = 0.97) [52]. The AARP was administered after the completion of the program.

#### 2.2.12. Treatment Evaluation Inventory-Short Form

Treatment acceptability was also assessed via the Treatment Evaluation Inventory-Short Form (TEI-SF) [54]. Parents were instructed to record their perception of treatment acceptability on a 5-point Likert scale. Sample items include “I believe this treatment is likely to be effective” and “I like the procedures used in this treatment” [54]. The total score was derived from the sum of all items, which involved reverse-scoring a negatively valenced item. Higher scores illustrate greater treatment acceptability, with a score of 27 or higher illustrating “moderate acceptability.” The TEI-SF has demonstrated good internal consistency (α = 0.85) [54]. The TEI-SF was administered after the completion of the program.

#### 2.2.13. Dyadic Parent Child Interaction System (DPICS-IV)

The Dyadic Parent Child Interaction System (DPICS-IV) is a standardized behavioral observation system used to assess parent-child interactions in children 2 to 7 years of age [55]. This observational coding system includes three different standardized situations to assess these interactions: Child-Led Play, Parent-Led Play, and Clean-up. Research assistants trained in the DPICS coding to an acceptable criterion (i.e., >80% agreement in the major DPICS categories) were the coders in the current study. One set of codes are provided in the results due to high interrater agreement (>80%).

### 2.3. Treatment

The intervention was provided by two clinical psychology doctoral students under the supervision of a licensed clinical psychologist who is a certified therapist and global trainer in PCIT. The doctoral students had completed the standard 40 h training for PCIT therapists and had received at least 2 years of supervision on PCIT cases. The students also received approximately 10 h of training on PCIT-Health in addition to previous supervision on at least two PCIT-Health cases before beginning treatment with the Johnson family. The Johnsons provided consent for participation in the program and use of their de-identified material for the present case study.

#### PCIT-Health Telehealth Intervention

PCIT-Health is an adaptation of PCIT that was developed to target the parent-child relationship, general parenting practices, and behavioral management in contexts salient to obesity (e.g., during mealtime, around screen time). Populations suited for this program are parents and their children 2 to 7 years of age who may be at risk for childhood obesity due to problematic feeding and/or screen time practices [56]. This adaptation maintains the core phases of PCIT (i.e., CDI and PDI) and adds a third phase, a health module, health-directed interaction (HDI). Similar to other PCIT adaptations [57,58], PCIT-Health is a session-limited program. Each phase begins with instruction in the respective skills before parents receive in vivo coaching in the use of the skills with their child. Each phase is 4 sessions in length (1 didactic session; 3 coaching sessions), for a total of 12 sessions. The program is focused on increasing rates of responsive caregiving within the context of developmentally appropriate limit-setting in both general and obesity salient situations (e.g., mealtime, screen time). Specifically, in the third module (i.e., HDI), parents are coached to generalize their child-centered and limit-setting skills when eating a meal or using a screen with their child. The goals of this module are to target obesity risks by promoting healthy food choices, providing instruction in effective feeding practices, and aiding parents to set effective limits on screen devices. For a more complete description of the program, see Niec et al. [59].

Although PCIT-Health began as an in-person program, due to the COVID-19 pandemic and social distancing guidelines, it was adapted for remote service delivery. The recent increase in the use of videoconferencing technology for mental health services, along with recommendations from previous research on I-PCIT, made it possible for PCIT-Health to be delivered online while remaining consistent with the original protocol.

Child-Directed Interaction (CDI). During the initial phase of the program, Child-Directed Interaction (CDI), the focus was on nurturing the parent–child relationship and reinforcing Ellen’s prosocial behaviors. Within this phase, the Johnsons were coached in increasing their child-centered skills (i.e., “Do Skills”) while decreasing their behaviors that take away Ellen’s lead of the play (i.e., “Don’t Skills”). During this phase, both parents reported concerns about Ellen’s attention-seeking behavior of whining and her lack of engagement in cooperative play (e.g., sharing toys). Throughout coaching, the therapists helped Mr. and Ms. Johnson praise Ellen’s use of an appropriate tone of voice to decrease her whining, while also using modeling and praises of sharing to increase her cooperative play. To help Ellen increase her ability to remain emotionally regulated when she became frustrated, the therapists began coaching the Johnsons in praising Ellen for remaining calm when an activity did not go her way (e.g., her tower fell over). While the therapists helped the Johnsons acquire these child-centered skills to foster responsive caregiving and prosocial child behaviors, they also began seeking out opportunities to shape Ellen’s health-related behaviors. They did this through imitating (e.g., “I want to eat healthy vegetables just like you”), describing food play (e.g., “You chose to make a healthy salad as your meal”), and praising positive child behaviors (“Thank you so much for sharing that healthy carrot with me!”). Mr. and Ms. Johnson started this initial phase by practicing the skills at home three or four times a week between sessions. However, they quickly increased their rate of practice to six or seven times a week, which they maintained over the course of the program.

Parent-Directed Interaction (PDI). In Parent-Directed Interaction (PDI), the second phase of the program, the Johnsons were instructed in and then coached through an effective and developmentally appropriate limit-setting sequence (i.e., the use of commands and time-out as outlined in the PCIT manual, 32). The therapists coached the Johnsons to introduce this new limit-setting sequence to Ellen using a stuffed animal (e.g., Mr. Bear) and practiced commands in simple play situations. As sessions progressed, therapists began coaching the Johnsons to give Ellen real-life commands (e.g., “Please come sit by me”) that could be generalized to health-related contexts (e.g., mealtime, screen time).

At the beginning of the PDI phase, Mr. and Ms. Johnson reported that Ellen was starting to sit at the dining table more consistently for meals and was becoming better at communicating and regulating her emotions. However, they also reported concerns surrounding lingering difficult behaviors, such as a lack of sharing and occasional aggressive play. Throughout the PDI phase, the therapists addressed these behaviors by continuing to coach Mr. and Ms. Johnson in their CDI skills (e.g., modeling calm, cooperative play; praising for sharing and playing calmly), while also coaching them in their newly learned PDI skills (e.g., giving direct commands to hand toys to parents, either to share or interfere with aggressive play). Given one of the Johnsons’ initial concerns involved Ellen cleaning up her toys, the therapists also coached both parents in real-life clean-up situations. By the end of the PDI phase, the Johnsons reported that Ellen was consistently helping clean up her toys, was frequently doing so without being asked, and was beginning to follow directions more consistently. As with CDI, Mr. and Ms. Johnson were completing 6 or 7 days of PDI and CDI homework each week.

Health-Directed Interaction (HDI). In the final phase, Health-Directed Interaction (HDI), the Johnsons were coached in generalizing their child-centered and limit-setting skills in the context of eating a snack/meal and using a screen device with Ellen. The Johnsons were excited to begin this final phase due to them having noticed some challenging behaviors around mealtime. For example, Mr. and Ms. Johnson reported that Ellen would often not finish her food, was picky when choosing foods, and was still having some difficulty staying seated at the dining table. To continue shaping Ellen’s health-related behaviors, the therapists coached Mr. and Ms. Johnson in both snack time and mealtime environments using healthy mealtime skills (e.g., praising healthy mealtime behaviors, allowing Ellen to make choices about her foods, recognizing when Ellen was full, etc.). Due to Ellen’s challenging mealtime behaviors of picky eating and getting up from the dining table, the therapists encouraged praising Ellen for trying unfamiliar foods (“Thank you for trying this new fruit”) and for remaining seated (“Thank you for staying seated at the table with us”). Mr. and Ms. Johnson also modeled trying unfamiliar/disliked foods and would describe to Ellen their taste and texture (e.g., “This cantaloupe is sweet and juicy.”). By the end of the HDI phase, the Johnsons were no longer pressuring Ellen to eat, Ellen was consistently sitting at the dining table for meals, and she was beginning to try unfamiliar foods.

In addition to the healthy mealtime skills, the therapists also provided instruction in the TIME strategies for managing Ellen’s use of screen devices (e.g., media should be Time-limited, parents should be Involved in media use, there should be Media free zones, and parents should prevent Exposure to inappropriate content.). During the live coaching session, the therapists coached the Johnsons in these TIME strategies while Ellen engaged with screen media. For example, Mr. and Ms. Johnson described the prosocial actions of the characters on the screen, provided transition statements when it was time to return to play, and praised Ellen for accepting limits when the device needed to be shut off. The therapists coached the Johnsons in generalizing the limit-setting skills to transition between screen time and playtime. By the end of the final phase, Mr. and Ms. Johnson reported limited challenges surrounding screen time as they did not allow Ellen to spend much time with devices.

## 3. Results

### 3.1. Treatment Progress

Changes were evident in the self-report scales completed by Mr. and Ms. Johnson (Table 1). From pre- to post-treatment, the Johnsons reported a reduction in total parenting stress and problematic feeding practices. They were less likely to pressure Ellen to eat or to restrict her intake of certain foods. They also engaged in less instrumental and emotional feeding. Further, the Johnsons perceived a reduction in Ellen’s disruptive behaviors, her negative lability, and her problematic media use from pre- to post-treatment. By contrast, the Johnsons perceived an increase in Ellen’s psychosocial competencies and emotion regulation and reported an increase in parental mediation of media use. Specifically, they increased their involvement with Ellen while she engaged with different types of media (e.g., pointed out prosocial behaviors of characters) and increased the restrictions placed on the type of media she consumed. Over the course of the program, the Johnsons reduced the amount of screen time that Ellen engaged in over the course of a week (e.g., from between 18 and 9 h at pre-treatment to between 10 h and 2 h and post-treatment; Table 1).

The Johnsons rated the PCIT-Health program as an acceptable and feasible prevention program and endorsed few barriers to treatment. On the AARP, both Mr. and Ms. Johnson indicated that they found the program highly acceptable. With each item being on a 6-point Likert scale (from 1 to 6) and higher items indicating increased acceptability, Mr. Johnson reported an average item score of 5.25 and Ms. Johnson reported an average score of 6. Similarly, on the TEI, the overall scores reported by both parents exceeded the cut-off for treatments being labeled as at least moderately acceptable. As measured by the BTPS, neither parent reported experiencing significant barriers to treatment, as they rated potential barriers as “never a problem” or “seldom a problem”.

In addition to the changes found in the self-report measures, changes occurred for the observational assessment, the DPICS-IV, suggesting the Johnsons experienced an increase in warm, responsive caregiving (Table 2). From pre- to post-treatment, Mr. and Ms. Johnson experienced a large increase in their “Do Skills” (i.e., behavior descriptions, reflections, and labeled praises) and a large reduction in their “Don’t Skills” (i.e., questions, commands, and criticisms). Most notably, Ms. Johnson saw a reduction from 73 questions to 0 in just three coaching sessions. Similarly, Mr. Johnson saw a decrease from 44 questions to 0.

**Figure 1:**
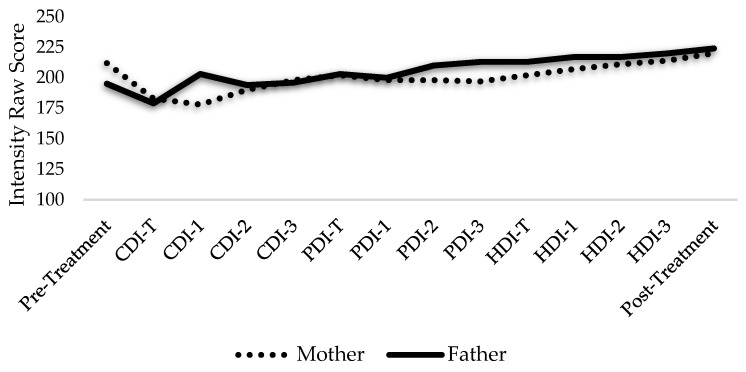
Parental Perception of Ellen’s Psychosocial Competence Throughout Treatment. Note: CDI = child-directed interaction; PDI = parent-directed interaction; HDI = health-directed interaction; T = teach session. The Johnsons’ perception of Ellen’s psychosocial competencies throughout the PATCH program as measured by the Psychosocial Strengths Inventory for Children and Adolescents.

**Figure 2:**
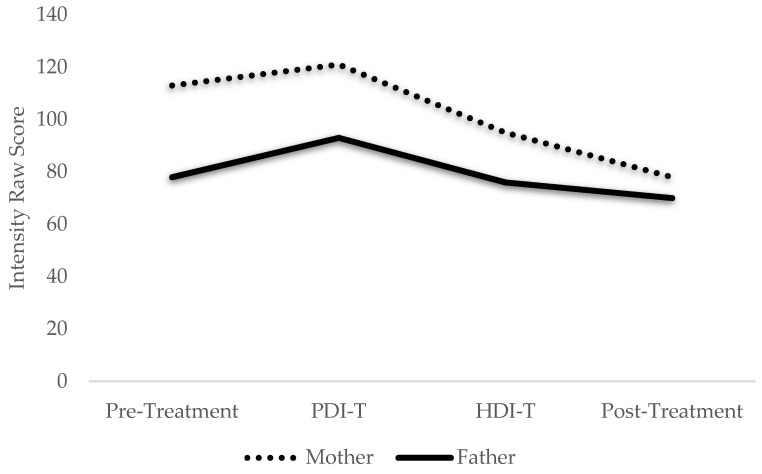
Parental Perception of Ellen’s Disruptive Behaviors Throughout Treatment. Note: PDI = parent-directed interaction; HDI = health-directed interaction; T = teach session. The Johnsons’ perception of Ellen’s disruptive behaviors at four time-points—pre-treatment, post-treatment, and at the transition of each phase as measured by the Eyberg Child Behavior Inventory.

**Figure 3:**
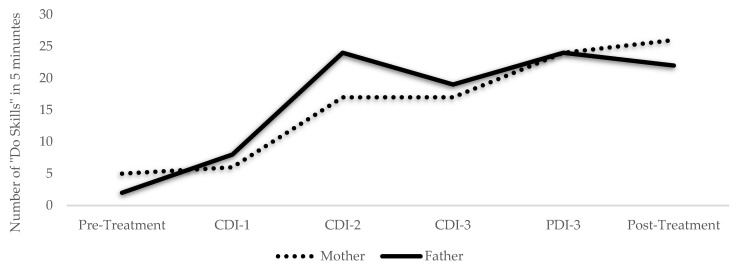
The Johnsons’ Use of the “Do Skills” Throughout Treatment. Note: CDI = child-directed interaction; PDI = parent-directed interaction; “Do Skills”: behavior descriptions, reflections, and labeled praises. The Johnsons’ use of “Do Skills” in a 5-minute coding throughout the PATCH program as measured by the Dyadic Parent–Child Interaction Coding System [55]. These were coded weekly by the doctoral student therapists; one set of codes is reported due to high inter-rater agreement (>80%).

**Figure 4:**
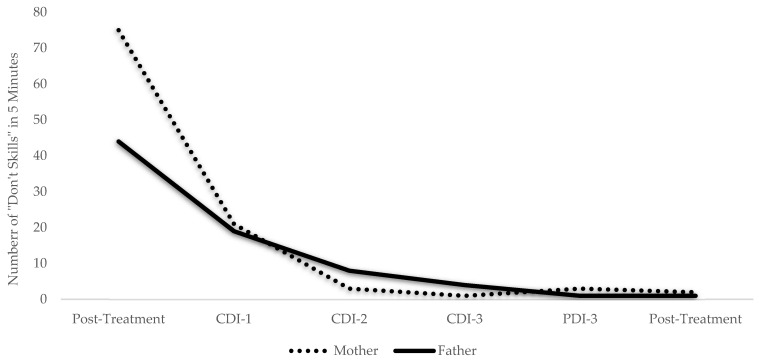
The Johnsons’ Use of the “Don’t Skills” Throughout Treatment. Note: CDI = child-directed interaction; PDI = parent-directed interaction; “Don’t Skills”: questions, commands, criticisms. The Johnsons’ use of the “Don’t Skills” in a 5-minute coding throughout the PATCH program as measured by the Dyadic Parent–Child Interaction Coding System [55]. These were coded weekly by the doctoral student therapists; one set of codes is reported due to high inter-rater agreement (>80%).

Within-treatment changes were also noted for the Johnsons. Weekly assessments of Ellen’s psychosocial competence revealed a steady increase from pre- to post-treatment (Figure 1). By contrast, assessments gathered after each phase revealed a reduction in the Johnsons’ perception of Ellen’s disruptive behaviors over the course of the program (Figure 2). Weekly measures of parent-child relationship quality revealed that while the Johnsons steadily increased their “Do Skills” (i.e., behavior descriptions, reflections, labeled praises) over the course of the program (Figure 3), and their “Don’t Skills” (i.e., questions, commands, and criticisms) were almost non-existent by post-treatment (Figure 4).

### 3.2. Strengths and Challenges of Telehealth

In addition to the general benefits associated with the telehealth modality (e.g., maintaining social distancing, reducing health disparities), the Johnson family demonstrated a great fit for receiving PCIT-Health virtually. First, the family had a lack of distractions in the home environment. Due to their family structure, the only individuals in the home were Ellen and Mr. and Ms. Johnson. This contrasted with many families who may have the additional challenge of managing other children or pets while attending virtual appointments. For families with additional siblings or other distractions (e.g., pets or other family members), therapists may find that the lack of control over the home environment to be a challenge with the telehealth modality [37]. Therefore, it is important that therapists first discuss with parents how to choose an appropriate area in the home for coaching (e.g., one that has minimal distractions, does not have a lot of options for the child to wander) and problem-solve with the family about what will happen when their child cannot be seen on the camera [60]. This challenge was especially evident when working with the Johnson family. Specifically, the Johnsons had chosen to complete sessions in their living room, a room with limited distractions and easy access to their technology (i.e., a desktop computer). However, given the open floor plan of the living room, there were multiple other rooms where Ellen could wander during coaching and, thus, was not able to be seen by the therapists on camera. Compared to in-person PCIT-Health delivery, the therapists used a substantial amount of coaching time encouraging Mr. and Ms. Johnson to use their skills to re-engage Ellen in the living room play. Clinicians should similarly use coaching time to facilitate engagement when distractions are hard to avoid in the family’s home environment.

The lack of visibility for details on the therapists’ computer screens was another notable challenge. Specifically, if Mr. and Ms. Johnson directed Ellen to hand them an object, it was difficult for the therapists to determine if Ellen was handing the requested object or a different one. During these instances, it was helpful to direct Mr. and Ms. Johnson to clearly point to the object they were requesting, while at other times, the therapists had to rely heavily on parental judgment of compliance. This differs from in-clinic PCIT-Health sessions, where judgments of compliance are often up to the therapist. The challenge of visibility was also present during the HDI phase when the therapists were unable to see or hear what was happening on the Johnsons’ mobile device used for in vivo coaching of parental mediation skills. This necessitated the therapists coaching Mr. and Ms. Johnson in thoroughly describing what was happening on the screen, which inadvertently increased their involvement in the screen activity. The Johnsons also demonstrated a good understanding of technology and its inevitable challenges. Due to the COVID-19 pandemic, it was not possible to help the family set up their telehealth technology in person, but Mr. and Ms. Johnson were flexible and willing to try out different device set-ups for coaching. This allowed the therapists to find an option that worked best for them while also ensuring adequate audio and visual quality. One of the most significant barriers that therapists may encounter when beginning to provide telehealth services is the accessibility of the necessary technology [37]. Essential components of online delivery include high-speed internet, a device with a webcam, and the ability to use the designated video conferencing platform. Helping the family with technology issues can take time out of sessions, while inadequate sound or video quality of devices can make in vivo coaching difficult [61]. I-PCIT researchers have recommended many strategies for managing these challenges including (1) scheduling a “Tech Session” at the start of treatment to ensure technology is set up appropriately, and (2) making a plan for how therapists will manage a session if technology problems interfere [60]. The therapists scheduled this session with the Johnsons prior to beginning the program and were able to test out the video conferencing platform and troubleshoot how technology issues would be handled in future appointments.

Finally, the Johnsons’ proximity to the clinic made it possible for food to be delivered to them during the HDI phase. Fortunately, they lived within the range of food delivery services, and the therapists could deliver fresh fruit and vegetables to them immediately before sessions. For families who live in remote or more rural regions, some creativity may be necessary to implement the Health-Directed Interaction, which requires in vivo coaching around snacks and mealtime. To deal with the challenges of getting food to families who received PCIT-Health via telehealth during the COVID-19 pandemic, therapists took extra steps to have appropriate food options delivered to families, so that they could continue to participate remotely. For example, some clinicians picked up grocery store orders and delivered the groceries to the family or dropped them off at the parents’ place of work. In other instances, clinicians discussed possible snacks/meals that the family already felt comfortable preparing and requested if these could be served during HDI sessions. Clinicians should collaborate with families to facilitate ease in access to foods suitable for HDI sessions.

## 4. Conclusions

Childhood obesity is a critical, yet preventable, public health concern that has been heightened by the COVID-19 pandemic and the subsequent stay-at-home orders. Since the onset of the pandemic, children have decreased their consumption of healthy foods [17] and increased their sedentary activity [19], both of which are risk factors for obesity. To mitigate these risks, PCIT-Health targets the parent–child relationship, general parenting practices, and behavioral management in contexts salient to obesity (e.g., mealtime, screen time).

Although the pandemic was associated with a multitude of challenges, one benefit was increased access to virtual mental health treatment. When adapting to a virtual format, several considerations needed to be addressed for successful intervention completion (e.g., technology concerns, food delivery/coordination).

As illustrated by the Johnson family, PCIT-Health has the potential to increase positive parent-child interactions, decrease challenging child behaviors, and foster healthy mealtime and screen time practices for parents and their children. Specifically, from pre- to post-treatment, the Johnsons exhibited an improvement in parenting skills (e.g., child-centered and directive) and reported a reduction in Ellen’s challenging child behaviors (e.g., defiance and lability). Similar improvements have been noted in other session-limited PCIT adaptations [56,57]. In addition, the Johnsons also exhibited improvements in healthy feeding practices (e.g., less restriction and pressure to eat; Ellen remaining seated at the table) from pre- to post-treatment. Previous research has documented the effectiveness of live coaching in the context of family mealtime interactions on increasing parents’ use of health-promoting behaviors and decreasing children’s problem behaviors during meals [62]. The Johnsons also reported healthier screen time practices such that they were more involved in Ellen’s media use and noted a reduction in the frequency of screen media use from pre- to post-treatment.

Similar to other case studies, there are limitations with the generalizability of these results. Because this was a case study, the results reported here provide a starting point from which to understand the potential advantages and disadvantages of implementing PCIT-Health in a telehealth format. The findings of a single case study cannot be taken as evidence for the broad generalizability of the intervention across families of different configurations and backgrounds. We have described the outcomes for one family whose many strengths helped support the success of the telehealth modality. For example, the Johnsons experienced limited difficulty with technology and had minimal distractions in the home environment. However, looking so closely at the challenges and strengths experienced by one family helps to create a picture of ways in which the model may or may not succeed with other families. That said, due to this case being a part of a larger RCT, we hope the results from the overall study will provide additional evidence for generalizability.

To address these limitations, future research should be conducted. Future research should first focus on evaluating the efficacy and effectiveness of the PCIT-Health telehealth model. To test the efficacy of the PCIT-Health model requires a randomized-control trial of the intervention with a large sample with demographic diversity. Subsequently, effectiveness studies should explore the transportability of the model into the community and the size of treatment effects when delivered by community-based therapists. Next-step studies should consider the key mechanisms of change in the intervention leading to positive outcomes.

Despite these limitations, this case study illustrates the potential of PCIT-Health in the prevention of obesogenic behaviors and reduction of disruptive behaviors. Given the challenges of the pandemic, the strategies provided by the PCIT-Health virtual model may be one potential buffer against the negative effects of COVID-19 on the behavioral health of children and families.

## Figures and Tables

**Table 1:** Survey Measures at Pre- and Post-Treatment.

Survey-Scale	Father		Mother	
	Pre-Treatment	Post-Treatment	Pre-Treatment	Post-Treatment
Parenting Practices				
CFQ—Pressuring to Eat	3.00	1.75	3.75	1.50
CFQ—Food Restriction	2.63	1.88	2.63	1.25
PFQ—Emotional Feeding	2.00	1.20	1.40	1.00
PFQ—Instrumental Feeding	2.50	1.50	1.50	1.25
PMS—Active Mediation	10.00	15.00	11.00	20.00
PMS—Restrictive Mediation	17.00	16.00	20.00	20.00
PMS—Social Co-Viewing	13.00	14.00	15.00	20.00
PSI—Total Stress	81.00	57.00	81.00	63.00
Child Functioning				
ECBI—Intensity	78.00	70.00	113.00	78.00
ERC—Negative Lability	31.00	24.00	31.00	27.00
ERC—Emotion Regulation	27.00	29.00	30.00	32.00
PMUM	2.00	1.22	1.67	1.11
PSICA—Intensity	195.00	224.00	212.00	220.00
Screen Time (hours/week)	18.00	10.00	9.00	2:00
Sleep (hours/night)	10.00	10.00	9.00	9:00

Note: CFQ = Child Feeding Questionnaire (range = 1–5); PFQ = Parental Feeding Questionnaire (range = 1–5); PMS = Parental Mediation Scale (range = 5–20); PSI = Parenting Stress Index (range = 36–180); ECBI = Eyberg Child Behavior Inventory (range = 36–252); ERC = Emotion Regulation Checklist (Negative Lability range = 16–64; Emotion Regulation range = 8–32); PMUM = Problematic Media Use Measure (range = 1–5); PSICA = Psychosocial Strengths Inventory for Children and Adolescents (range = 36–252).

**Table 2:** Observational Measure at Pre- and Post-Treatment.

DPICS-IV Category	Father		Mother	
	Pre-Treatment	Post-Treatment	Pre-Treatment	Post-Treatment
Do Skills				
Behavior Descriptions	0	8	0	5
Reflections	2	4	5	8
Labeled Praises	0	10	0	13
Unlabeled Praises	2	0	6	5
Don’t Skills				
Questions	40	0	73	0
Commands	4	1	2	2
Criticisms	0	0	0	0

Note: DPICS-IV = Dyadic Parent–Child Interaction Coding System, Fourth Edition.

## Data Availability

The data presented in this study are unavailable due to client confidentiality.
